# Study of the Incorporation of Gel and *Aloe vera* Peel Extract in a Polymer Matrix Based on Polyvinylpyrrolidone

**DOI:** 10.3390/polym16141998

**Published:** 2024-07-12

**Authors:** Britania Janet Gutiérrez Rafael, Orlando Zaca Moran, Raúl Jacobo Delgado Macuil, Hugo Martínez Gutiérrez, Marcos García Juárez, Valentin Lopez Gayou

**Affiliations:** 1 Departamento de Nanobiotecnología y Biosensores, Centro de Investigación en Biotecnología Aplicada, Instituto Politécnico Nacional (IPN-CIBA), Santa Inés Tecuexcomac 90700, Tlaxcala, Mexico; bgutierrezr1800@alumno.ipn.mx (B.J.G.R.); ozacam@ipn.mx (O.Z.M.); rdelgadom@ipn.mx (R.J.D.M.); 2 Centro de Nanociencias y Micro y Nanotecnologías CNMN IPN, Av. Luis Enrique Erro s/n, Nueva Industrial Vallejo, Gustavo A. Madero, Ciudad de México 07738, Mexico; humartinez@ipn.mx; 3 Centro de Investigación en Reproducción Animal, Universidad Autónoma de Tlaxcala-CINVESTAV, Plaza Hidalgo Ote. 9, Cuarto Barrio, Panotla 90140, Tlaxcala, Mexico; garcia_juarez_marcos@yahoo.com.mx

**Keywords:** membranes, *Aloe vera*, polyvinylpyrrolidone (PVP), electrospinning

## Abstract

The development of dressings based on electrospun membranes with polymers and plant extracts is an interesting approach to skin regeneration, providing elements to prevent contamination and a matrix that accelerates the healing process. We developed a membrane composed of polyvinylpyrrolidone (PVP), gel and *Aloe vera* peel extract via the electrospinning technique. Additionally, an optimal ratio of PVP/*Av* gel/*Av* skin extract was determined to facilitate membrane formation. Electrospun membranes were obtained with fiber diameters of 1403 ± 57.4 nm for the PVP and 189.2 ± 11.4 nm for PVP/*Av* gel/*Av* peel extract, confirming that the use of extracts generally reduced the fiber diameter. The incorporation of gel and peel extract of Aloe vera into the electrospun membrane was analyzed via FTIR and UV–Vis spectroscopies. FTIR revealed the presence of functional groups associated with phenolic compounds such as aloin, aloe-emodin, emodin and aloesin, which was confirmed by UV–Vis, revealing absorption bands corresponding to aloin, phenols and carbonyl groups. This finding provides evidence of the effective integration and prevalence of bioactive compounds of a phenolic and polysaccharide nature from the gel and the *Av* skin extract in the electrospun fibers, resulting in an advanced membrane that could improve and accelerate the healing process and protect the wound from bacterial infections.

## 1. Introduction

*Aloe vera* (*Av*) is considered the most bioactive plant among all 420 species of Aloe. It is described as a xerophytic perennial succulent plant resembling a cactus, belonging to the *Aloe* genus, known for its high adaptability and versatility, which allow it to survive in dry and hot environmental conditions [1]. *Av* is one of the species receiving significant attention in tissue engineering due to its effectiveness in the wound healing process through various mechanisms like wound moisture retention, the promotion of cell migration, the stimulation of collagen production, and a reduction in inflammation [2,3].

Morphologically, *Av* leaves are composed of two main structures. The first is known as peel or epicarp, from which extracts are usually taken for medicinal applications, given that it has a wide variety of compounds of a phenolic nature, mainly anthraquinones C-glycosides, anthrones and free anthraquinones such as Aloin [4]. When the peel is separated from the gel, there are also residues of the latex found between these two elements that have high contents of C-glycosylchromones and anthrones [5]. Normally, the peel is discarded when separating it from the gel, since the latter is the product with high commercial value. However, the high content of bioactive compounds with antibacterial activity that are present in the *Av* peel could lead to its use in medical applications.

The gel or parenchyma, which is the most utilized component, located on the inner part of the leaves, is mainly composed of 99–99.5% water [6], with the remaining percentage consisting of phytochemicals, including polysaccharides, enzymes, hormones, vitamins and flavonoids, among others [7]. It is due to the presence of these compounds and their properties that the gel can be integrated into a matrix for tissue applications that require biocompatibility, biodegradability and specific mechanical characteristics [8,9].

Modern dressings such as films, foams, hydrocolloids and hydrogels offer advantages such as the ability to store and release molecules, low adhesion to the wound, the ability to act as a barrier and the ability to obstruct the formation of epithelial tissue [10]. Membranes manufactured via the electrospinning technique possess ideal characteristics for use as supports, as they can be functionalized via the incorporation of materials that lack the necessary mechanical properties to be spun into a polymer [11].

In electrospinning, a high-voltage source connected to a stainless-steel needle and the collector generates an electric field that causes the polymer solution loaded in the syringe to be expelled, generating filaments that are deposited in the form of nanometric fibers on a collector. These fibers accumulate and are arranged to form a membrane with a high surface/volume ratio, which makes it ideal for the treatment of wounds [12] as it can mimic the architecture of the skin’s extracellular matrix, allowing for the interaction of membranes and materials, as well as perspiration in the wound [13,14].

Polyvinylpyrrolidone (PVP) is a non-toxic synthetic polymer, which is approved by the FDA as a drug delivery system due to its physicochemical and mechanical characteristics [15]. Structurally, it possesses a strong hydrophilic fraction, pyrrolidone and a hydrophobic fraction due to its alkyl group. These characteristics enable it to interact with different cellular components [16,17]. PVP finds applications in the pharmaceutical, biomedical and food industries [18,19].

Several studies have been conducted on the incorporation of medicinal plant extracts into polymeric matrices for antibacterial and wound healing applications [3]. Attempts have been made to integrate *Av* into electrospun polymers, such as the use of polycaprolactone (PCL) with varying percentages of *Av* gel powder (0%, 5%, 10% and 15%) in a chloroform–methanol solution, resulting in membranes that exhibited bead formation [20]. Likewise, the use of PVP as polymer on electrospun membranes has been previously investigated, such as the characterization of membranes composed of PVP (1 mm ± 0.2 μm), propolis and *Av* powder using physiochemical methods [21]. Moreover, manufactured electrospun membranes of PVP with *Av* and acetylated *Av* demonstrated that changes in the components of the extracts used for the functionalization of the membranes have an impact on mechanical and microstructural properties and antibacterial activity against fungi and bacteria [22]. However, in most of the works aiming to incorporate *Av* into membranes, multiple polymers are mixed, necessitating the use of different solvents.

While studies have investigated the incorporation of *Av* gel conjugated with various polymers into electrospun fibers, there remains a lack of information regarding potential losses or alterations in compounds obtained using freeze-dried extracts that were subjected to the electrospinning process. Specifically, further research is needed to explore the additional inclusion of *Av* peel extract, which is often discarded despite containing numerous compounds with antibacterial activity. In this study, PVP membranes were manufactured with peel extract and *Av* gel incorporated into PVP membranes produced via the electrospinning technique. The objective was to investigate the incorporation and effect of peel extract and *Av* gel percentages on the membrane’s architecture. Despite the decrease in the fibers’ diameter upon the incorporation of the plant’s bioactive components, the *Av* compounds remained present and available during electrospinning, making them ideal for applications in wound healing.

## 2. Materials and Methods

### 2.1. Obtaining Biological Material

The leaves of *Av* (*Aloe barbadensis* Miller, AV) with two years of growth were obtained from the community of Huejotzingo Puebla, Mexico.

The chemical materials that were used were of analytical grade. Polyvinylpyrrolidone (PVP) with a molecular weight of 360,000 g/mol was obtained from the company Sigma-Aldrich (Merck KGaA, Darmstadt, Germany). Ethanol (cat 0390) and methanol (cat 5420) of analytical grade were purchased from Meyer (Ciudad de México, México). Deionized water and distilled water were obtained from a model 90750 water/pro/RO purifier (Labconco, Kansas City, KS, USA) that was used for washing materials and preparing solutions.

### 2.2. Obtaining of Peel extract and Aloe vera Gel

The *Av* leaves were washed with running water to remove any residue. Subsequently, they were rinsed with distilled water. The gel was extracted from the middle part in the direction of growth, as well as the peel. Both were washed and immersed in deionized water to prevent oxidation. The gel was then extruded with a cotton cloth to remove the fibers from the liquid part, and then the gel was centrifuged at 5000 rpm for 5 min. The whole leaf was cut into pieces and placed in a solution of ethanol and water (1:1), which was heated to 60 °C for three hours. Once this time had passed, the ethanol was removed and reduced in a rotary evaporator. Both components were lyophilized, ground and stored at 4 °C for later use.

### 2.3. Thin-Layer Chromatography (TLC)

The plant extracts were dissolved in methanol at a concentration of 1 mg/mL. The preparations were placed on TLC Silica gel 60 F254 (Merk, Darmstadt, Germany) chromatography plates, using ethyl acetate–methanol–water (1:1.35:0.1) as mobile phase. The Komarowasky reagent was used to reveal the compounds present in both extracts. The Bornträger reagent was used to visualize the anthracene-derived compounds in the *Av* gel samples to calculate the Retention factor (Rf). Chromatographic zones were visualized using ultraviolet light at 254 and 365 nm.

### 2.4. High-Performance Liquid Chromatography (HPLC)

To identify the chemical compounds of intermediate polarity contained in the whole leaf extract and *Av* gel, the extract was analyzed by HPLC. The analysis was carried out on a Waters 2695 liquid chromatograph equipped with a Waters 2996 photodiode array detector. The separation was carried out using an RP C-18 Superspher (Merk) column (120 × 4 mm; 5 µm) with the following ratios: for the mobile phase, solvent A was HPLC quality water plus 0.5% and solvent B was acetonitrile: A:B. The detection wavelength was recorded as 257–330 nm [23].

### 2.5. Preparation of Electrospun Polyvinylpyrrolidone and Aloe vera Membranes

Different preparations of PVP and PVP were made with the whole leaf and *Av* gel. To evaluate the effect of the flow rate on the fabrication of the membranes, PVP was prepared at 7% *w*/*v* in ethanol. On the other hand, three different ratios of PVP/*Av* gel and PVP/*Av* whole leaf extract were prepared at 8:2, 6:4 and 4:6% *w*/*v*. It is important to mention that the volume weight percentage of the whole leaf extract, and the lyophilized gel were dissolved in ethanol and stirred for 3 h; subsequently, the insoluble fraction was removed by centrifugation at 5000 rpm for 10 min. The final volume was measured to maintain the polymer/plant extract ratio by incorporating the PVP in each of the ratios to obtain homogeneous solutions. The membranes were fabricated in an electrospinning equipment consisting of a double-syringe infusion pump (KD Scientific, Holliston, MA, USA), a needle with an internal diameter of 0.51 mm, a high-voltage source with an operating range of 1–30 kV (Spellman, Bronx, NY, USA) and a 5 cm diameter circular copper collector plate coated with aluminum for each membrane. The operational conditions of the process consisted of a deposition time in the collector of 10 min, the distance from the infuser to the collector was 15 cm and the feed flow for the first test was 0.1 mL/h and 0.2 mL/h (Table 1). For PVP preparations, the whole peel extract and *Av* gel were set at 0.1 mL/h. The process was carried out under ambient temperature and humidity conditions.

### 2.6. Scanning Electron Microscopy (SEM)

The diameter and structural morphology of the membranes were characterized using a VEGA TS 5136SB microscope (TESCAN, Brno-Kohoutovice, Czech Republic) with a high-vacuum resolution (0.009 Pa) of 3.5 nm, secondary electron (SE) and backscattered (BSE) detectors, and a magnification range of 4–500,000× and an accelerating voltage of 0.5–30 kV. A JEOL model 7800F microscope with a high-resolution field emitter with a backscattered electron detector (BSE) was also used (JEOL, Tokyo, Japan). The analysis of the images was carried out using ImageJ program (https://imagej.net/ij/, accessed on 5 June 2024).

### 2.7. UV–Vis Spectrophotometry (UV–Vis)

The absorbance spectra of the peel extract and *Av* gel, as well as those of the electrospun membranes from PVP and *Av*, were obtained via spectrophotometer Genesys 10S UV–Vis, ThermoScientific (ThermoScientific, Shanghai, China), using the wavelength range from 190 to 800 nm, with a resolution of 2 nm. All samples were previously dissolved in ethanol for analysis.

### 2.8. Fourier Transform Infrared Spectroscopy (FTIR)

All elements utilized in the fabrication of the electrospun membranes underwent analysis using a Bruker Vertex 70 FTIR spectrometer (Bruker Optics Corporation, Billerica, MA, USA) operating in Fourier transform mode with ATR. Measurements were conducted within the mid-infrared spectral region (4000–400 cm^−1^) at a resolution of 4 cm^−1^ and an integration time of 60 s (1 s/scan). Data acquisition and processing were carried out using OPUS software, version 6.0 (Bruker Optics, USA).

## 3. Results and Discussion

### 3.1. Characterization of Aloe vera Components (Gel and Peel Extract)

**Figure 1:**
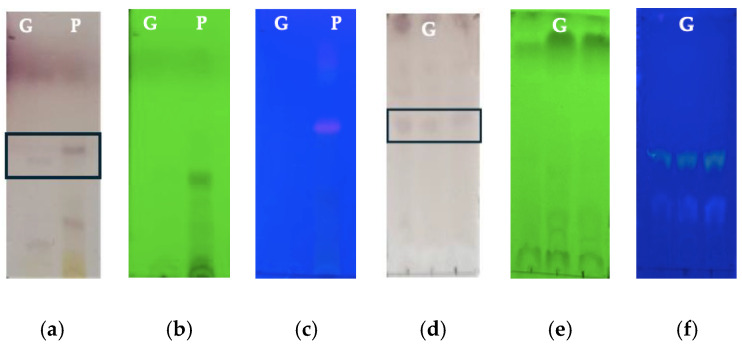
TLC in normal phase of *Av* gel (**G**) and *Av* peel extract (**P**): (**a**) Komarowasky in visible light; (**b**) 254 nm; (**c**) 365 nm; (**d**) Bornträger in visible light; (**e**) 254 nm; (**f**) 365 nm.

The TLC analysis carried out with the methanolic peel extract and *Av* gel showed the presence of compounds derived from anthraquinones, mainly Aloin, Aloe Emodin and Aloesinosides, with an Rf of 0.6 in the development with Komarowsky and an Rf of 0.25 with the Bornträger developer (spots boxed in the Figure 1a,d), as well as the presence of flavonols and flavonoids that showed fluorescence. It has been stated that compounds derived from anthracene quench fluorescence when irradiated at 254 nm, as seen in Figure 1b,e [24,25]. The intense orange point and the yellow point under irradiation at 365 nm in Figure 1c and Figure 1f, respectively, confirm the presence of aloin and anthracene derivatives in the peel extract and *Av* gel after the processing and obtaining the freeze-dried ones [26].

**Figure 2:**
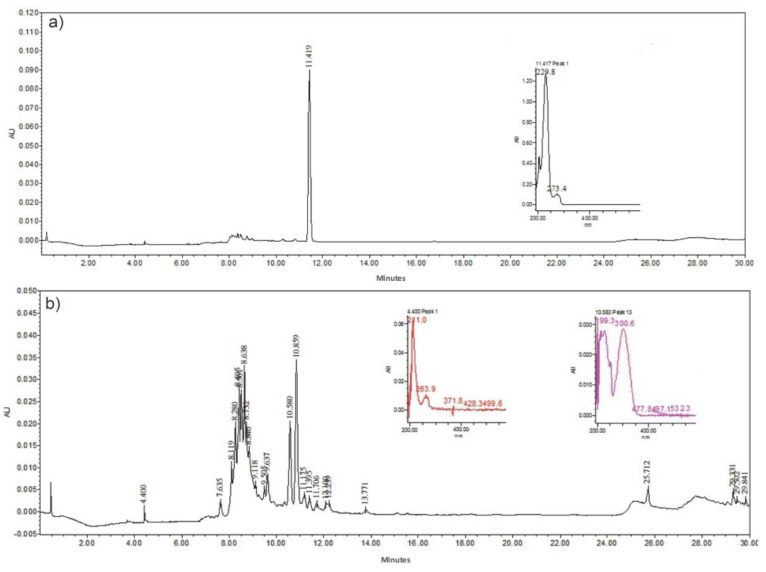
HPLC chromatograms and UV spectra: (**a**) *Aloe vera* gel and (**b**) *Aloe vera* peel extract.

The HPLC chromatograms of the peel extract and *Av* gel are observed in Figure 2. For the *Av* gel, the main identified compound was aloin, with a retention time (Rt) of 11.41 min and with an absorption spectrum in UV λmax of 229 nm. For the fraction obtained as the *Av* peel extract, a greater number of phenolic compounds, which were classified into four groups—phenolic acids, flavonoids, chromones and the anthrones Aloesin (aloeresin B), cis 5-p-Coumaroylquinic acid, Aloin B (isobarbaloin), Aloin A (barbaloin) and Malonyl aloin A) [27,28,29]. Throughout the process of obtaining the gel and *Av* peel extract, it is feasible to primarily preserve phenolic compounds, particularly those originating from anthracene, which are prone to degradation at each stage of plant processing. Compounds like Aloin B (isobarbaloin) and Aloin A (barbaloin), derived from anthracene, have been documented to have anti-inflammatory, antioxidant and antibacterial properties [30]. Hence, their incorporation into materials intended for wound treatment applications is critically significant.

**Figure 3:**
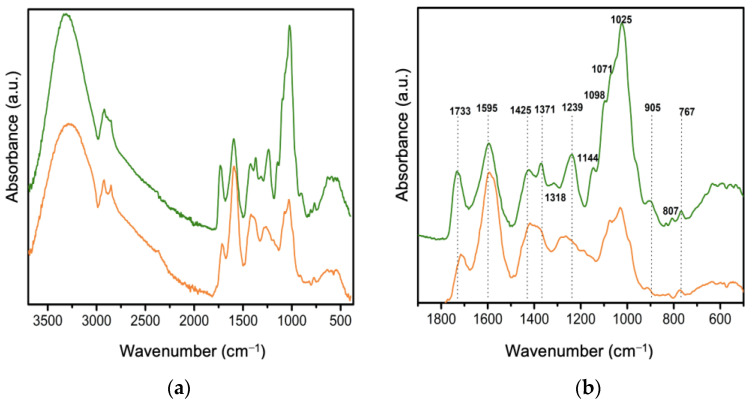
FTIR spectra of peel extract (orange) and *Av* gel (green): (**a**) 4000–400 cm^−1^ region and (**b**) 1900–500 cm^−1^ region.

Figure 3a shows the FTIR spectra of lyophilized gel and *Av* peel extract powders. In the 3500–2750 cm^−1^ range, both samples exhibit a prominent band attributed to the hydroxyl groups found in phenolic compounds like anthraquinones [31,32,33].

Figure 3b displays multiple bands in the 1900–500 cm^−1^ region, with higher intensity in the *Av* gel (1740, 1595, 1371 and 1239 cm^−1^). These bands correspond to C=C stretching, the symmetric COO stretching of carboxylates and the acetylation of acemannan, the main polysaccharide in *Av* gel [34].

Specifically, the band at 1595 cm^−1^, corresponding to benzene ring vibrations, is associated with compounds derived from anthracene such as aloin, aloe–emodin, emodin and aloesin. This band is predominantly observed in the whole leaf extract [33], confirming the nature of compounds obtained from both parts of the plant, as previously observed in TLC and HPLC analyses.

While anthraquinone compounds are predominant in the peel extract due to the processing of latex during production, FTIR analysis reveals a diverse array of bands in the carbohydrate fingerprint of *Av* gel. These include alcoholic, phenolic and acidic OH groups and carboxylic groups characteristic of aldehydes, ketones or acids [35,36,37]. The shoulder at 1071 cm^−1^ relates to C-O stretching in rhamnogalacturonan and galactose units [38], while the strong band at 1025 cm^−1^ corresponds to C-O and C-OH bonds in glucan polysaccharides found in both gel and *Av* peel extract [39]. Bands at 1076 and 1036 cm^−1^ indicate the presence of other sugars, such as mannose and glucose [31], and the 807 cm^−1^ band is associated with mannose absorption of a monosaccharide visible in the spectra of both the gel and *Av* peel extract [40].

Some of the identified groups belong to compounds that present with healing and antibacterial activity [41], which have been described and identified by other authors in the gel and *Av* peel extract. One of these compounds is mannan; this polysaccharide has been identified as the major bioactive component in the *Av* gel, presenting with healing activity [42]. Other carbohydrates in *Av* gel also have healing activity in addition to promoting cell proliferation [43]. The incorporation of the peel extract and *Av* gel compounds into electrospun membranes would allow their functionality to be enhanced by conferring healing and antibacterial activity.

### 3.2. Evaluation of Electrospinning Conditions to Obtain PVP Membranes

**Figure 4:**
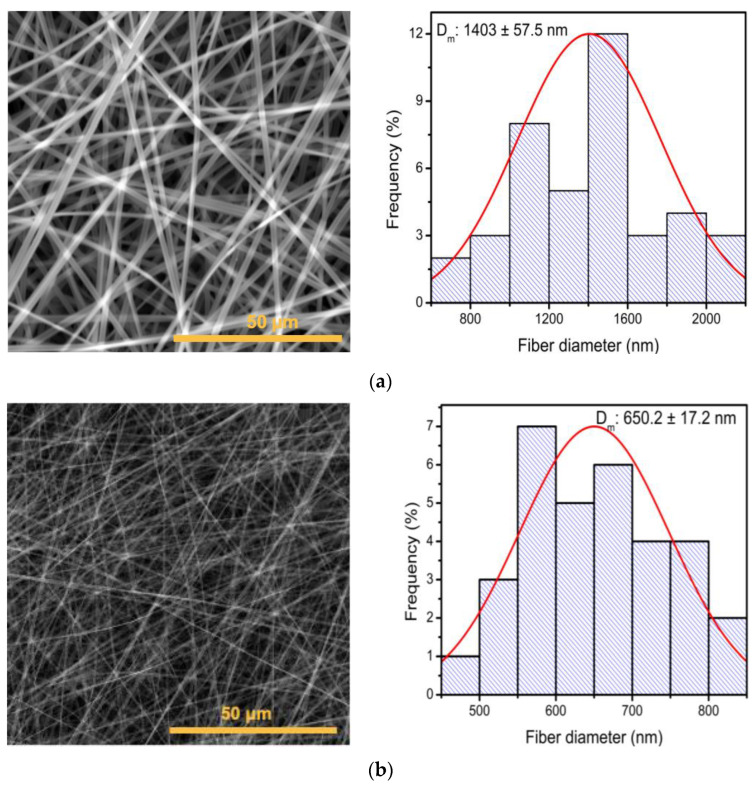
SEM micrograph of electrospun PVP membranes and fiber size distribution histogram: (**a**) 15 kV and a flow rate of 0.2 mL/h and (**b**) 20 kV and a flow rate of 0.1 mL/h.

The SEM study of the electrospun PVP membranes under condition A, using a voltage of 15 kV and a flow rate of 0.2 mL/h, exhibited membranes with a long, smooth and continuous morphology without bead formation, as illustrated in Figure 4a, and with an average diameter of 1403 ± 57.5 nm. On the other hand, the membranes obtained under condition B using a voltage of 15 kV and a flow rate of 0.1 mL/h presented with a decrease in diameter of 650.2 ± 17.2 nm (Figure 4b), reducing the diameter size of the fibers by 53.6%. Among the parameters that directly influence the fabrication of electrospun membranes, the flow rate directly affects the diameter size of the membranes [44].

The nanometric size of the fibers allows for a larger contact surface, which is a desirable characteristic in tissue engineering because it facilitates the interaction of the bioactive constituents integrated in the membranes on the extracellular matrix [32,45,46,47,48].

For the following analyses, evaluating the polymer/*Aloe vera* relationship, the conditions of 15 kV and 0.1 mL/h were selected for the manufacture of the membranes.

**Figure 5:**
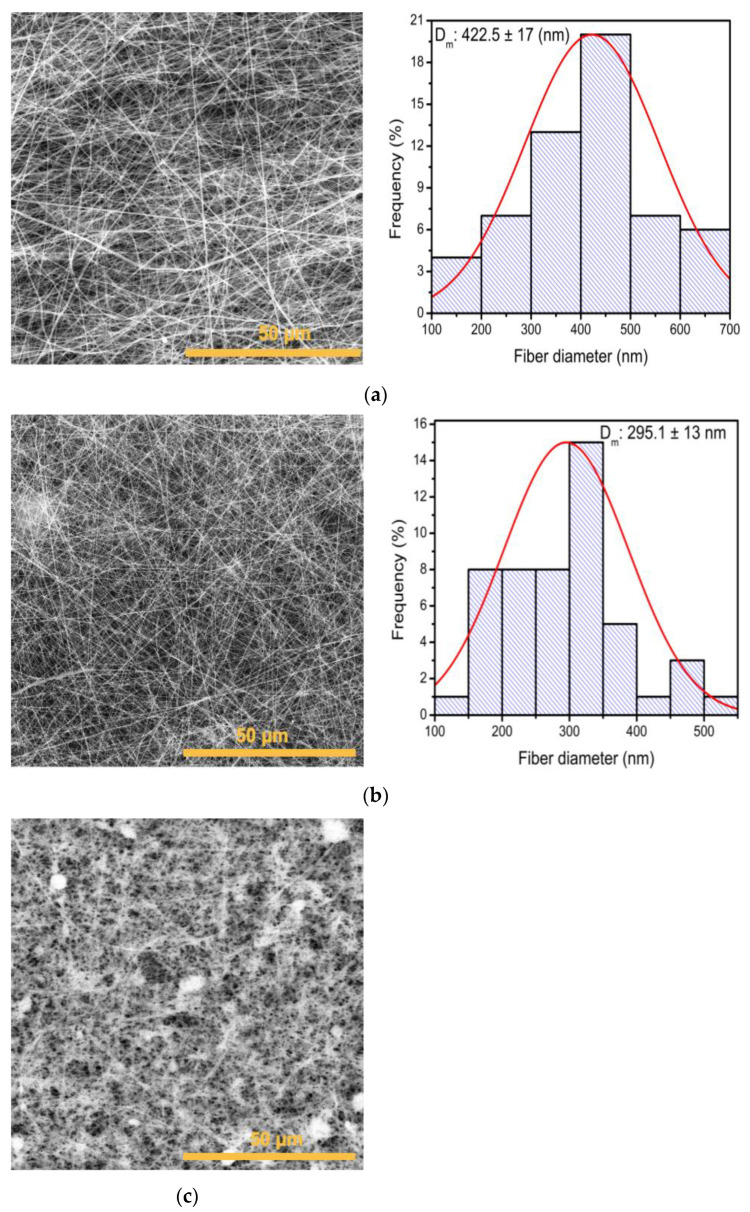
SEM micrograph of membranes of electrospun PVP/*Av* gel membranes and fiber size distribution histogram: (**a**) 8:2, (**b**) 6:4 and (**c**) 4:6 ratio.

Three proportions of PVP/*Av* gel were evaluated to determine which PVP/*Av* gel ratio allowed for the formation of fibers with the smallest diameter. The PVP/*Av* gel ratio that allowed for the formation of smaller-diameter nanofibers was 6:4 with a diameter of 295.1 ± 13 nm (Figure 5b), while, with the 8:2 ratio, fibers of 422.5 ± 17 nm were obtained (Figure 5a). On the other hand, with the 4:6 ratio, no fibers were deposited due to the lower polymer concentration that did not allow for the formation of threads, although organic material was present in the *Av* extract (Figure 5c).

**Figure 6:**
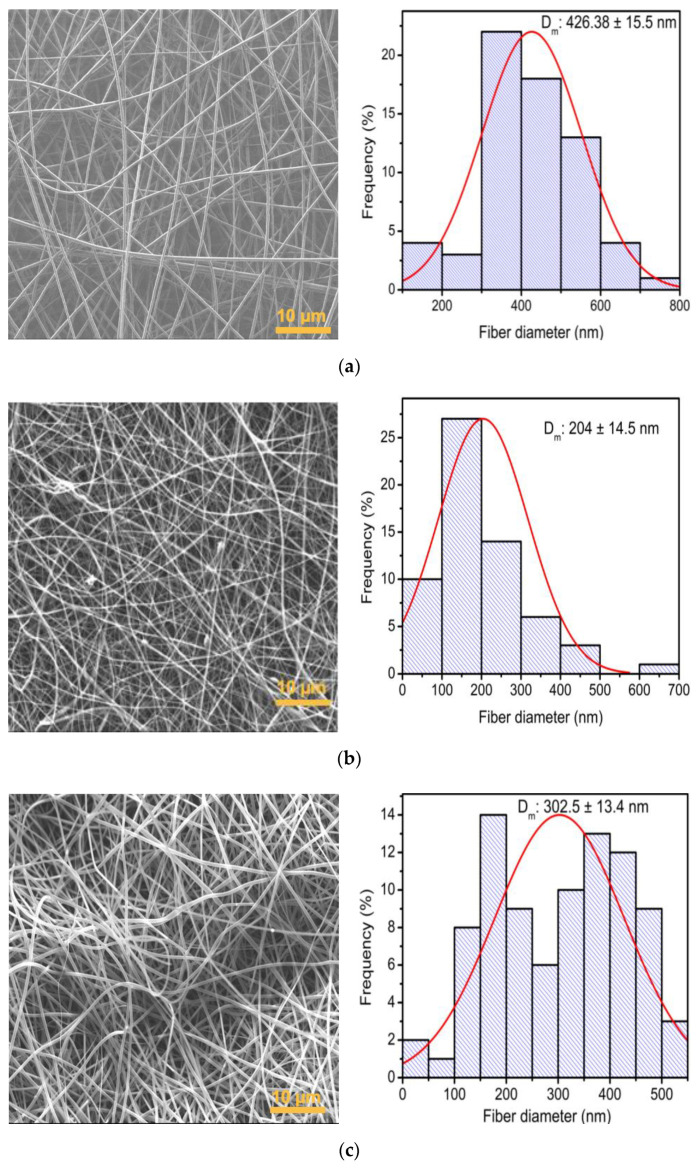
SEM micrograph of membranes of electrospun PVP/*Av* peel extract membranes and fiber size distribution histogram: (**a**) 8:2, (**b**) 6:4 and (**c**) 4:6 ratio.

The membranes obtained by combining PVP and *Av* peel extract are depicted in Figure 5. The fibers in the ratios 8:2, 6:4 and 4:6 were randomly distributed and had larger pores compared to membranes containing *Av* gel. Membranes in Figure 6a,c retained a smooth cylindrical shape with no visible beads along the fibers with sizes of 426.38 ± 15.5 nm and 302.5 ± 3.4 nm, respectively. However, in the 6:4 ratio (Figure 6b), beads suggesting the encapsulation of compounds were observed [47]. These membranes exhibited a distinct architectural change, characterized by fibers (204 ± 14.5 nm) with curvatures rather than the straight, perpendicular arrangement seen in PVP membranes (Figure 4a,b) and PVP with Aloe vera gel (Figure 5a–c).

**Figure 7:**
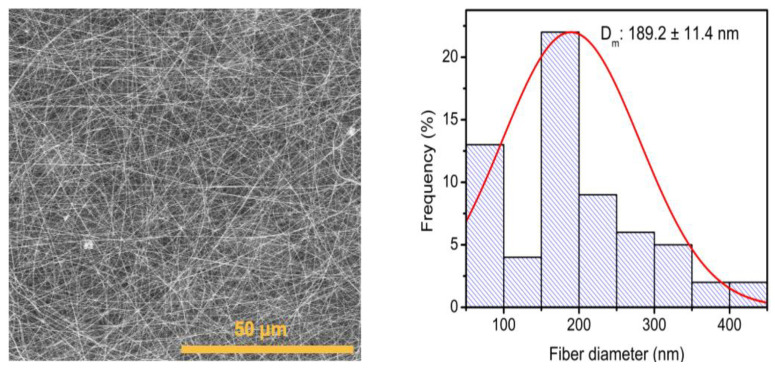
SEM micrograph of membranes of electrospun PVP/*Av* membranes (peel extract and gel with a 7:1.5:1.5 ratio) and fiber size distribution histogram.

Additionally, the incorporation of the *Av* peel extract into the mixture was evaluated, using a proportion of 8:1.5:1.5 PVP/*Av* gel/*Av* peel extract, with which a fiber diameter of 189.2 ± 11.4 nm was obtained (Figure 7), smaller than that obtained with the 6:8 PVP/gel ratio (Figure 5b). Because the *Av* peel extract comprised different compounds that solubilize in polar solvents, its addition to the polymer mixture did not increase the viscosity.

This is confirmed by a decrease in the diameter of the membranes, which is evidence that the incorporation of *Av* peel extract contributed to the decrease in membrane diameter. Therefore, this proportion was selected for the incorporation of the *Av* peel extract for subsequent analyses. 

### 3.3. Characterization of Electrospun Membranes from PVP/Av Gel by FTIR-ATR and UV–Vis

**Figure 8:**
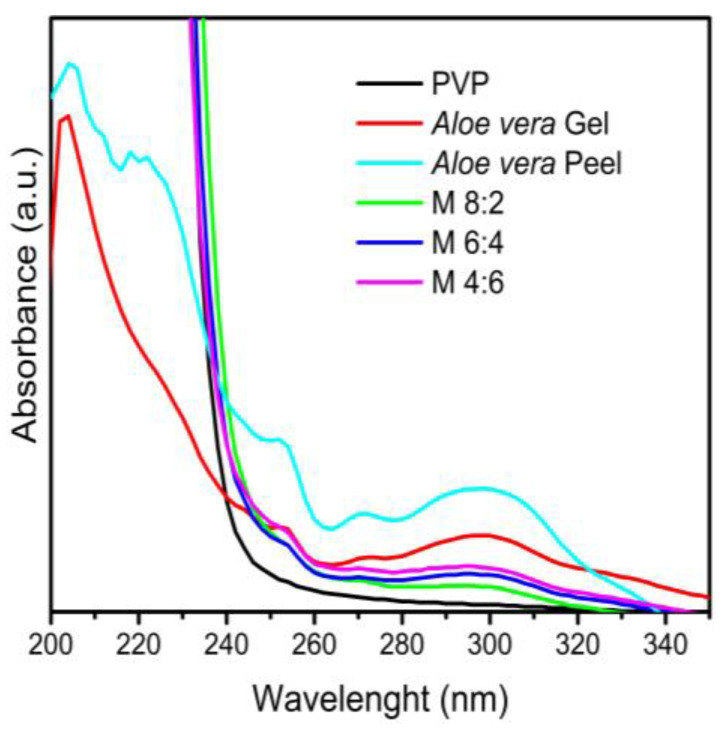
UV–Vis spectrum of PVP, gel and *Av* peel extract.

To confirm the incorporation of *Av* compounds into the electrospun membranes, UV–Vis and FTIR-ATR characterizations were carried out. The PVP, gel and *Av* peel extract, along with the electrospun membranes at three different PVP/*Av* gel ratios (8:2, 6:4 and 4:6), were analyzed using UV spectrophotometry (Figure 8).

Absorption bands at 280 nm corresponding to aloin, an anthraquinone compound present in both the gel and the Av peel extract, as well as phenolic bands at 320 and 330 nm, were identified [49,50,51].

These bands were consistently observed in the three PVP/*Av* gel membranes, with increased intensity correlating to higher concentrations of gel in the membrane, demonstrating the effective incorporation of *Av* gel compounds into the PVP electrospun membranes.

The presence of these bands in both lyophilized extracts and electrospun membranes suggests that the interactions between these compounds are likely non-covalent, involving physical inclusion or adsorption. This is supported by the separation observed in an aqueous medium between PVP and the bioactive compounds, highlighting the intrinsic absorption properties of each component within the electrospun membrane.

**Figure 9:**
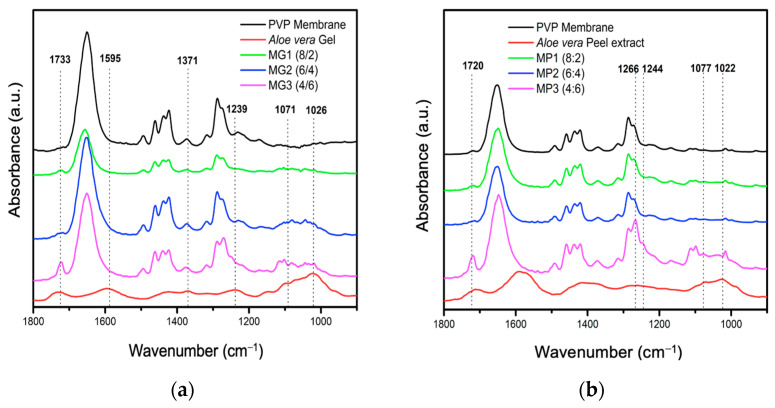
FTIR-ATR spectra: (**a**) PVP membrane and *Av* gel and (**b**) PVP membrane and *Av* peel extract (1800–900 cm^−1^).

The FTIR-ATR spectra of the electrospun membranes in the 8:2, 6:4 and 4:6 ratios of PVP/*Av* gel and PVP/*Av* peel extract, along with PVP membranes and lyophilized extracts, were compared (Figure 9a,b). The spectrum of the PVP membrane exhibited characteristic bands, notably a prominent band at 1646 cm^−1^ corresponding to C=O amide stretching, C-N stretching at 1463 cm^−1^ and CH_2_ bending at 1381 cm^−1^ in the pyrrolidone ring [52]. Additionally, a band at 1733 cm^−1^ was detected, which intensified with the increasing concentration of the lyophilized extracts, which was particularly noticeable in the 4:6 ratio of both electrospun membranes (PVP/*Av* gel and PVP/Av peel extract).

The spectra of the electrospun membranes in the 8:2, 6:4 and 4:6 ratios of PVP/*Av* gel exhibited characteristic carbohydrate bands at 1733 cm^−1^, 1595 cm^−1^, 1370 cm^−1^, 1244 cm^−1^, 1077 cm^−1^ and 1017 cm^−1^. These bands, coupled with the results from the UV–Vis characterization, provide evidence for the effective incorporation of *Av* compounds into the electrospun membranes.

The addition of *Av* peel extract to the PVP/*Av* gel mixture led to the formation of membranes with a smaller diameter than those made solely with PVP/*Av* gel. The membrane derived from the PVP/*Av* gel/*Av* peel extract mixture (8:1.5:1.5) was characterized using ATR-FTIR to confirm the incorporation of both the gel and the *Av* peel extract constituents into the electrospun membranes.

**Figure 10:**
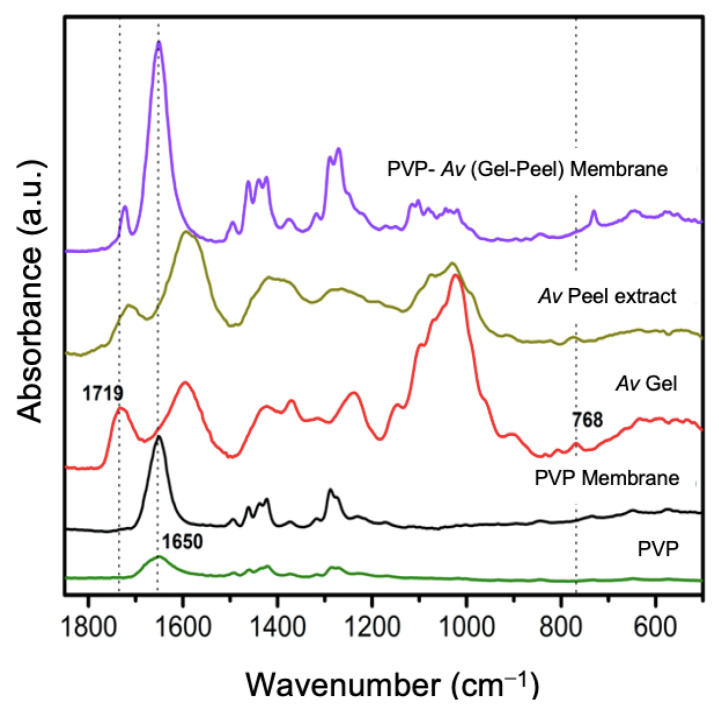
FTIR spectra of PVP powder, PVP membrane, gel and *Av* peel extract (1850–550 cm^−1^).

The FTIR-ATR spectra comparison of the PVP/*Av* gel/*Av* peel extract membrane (8:1.5:1.5) with a membrane composed exclusively of PVP, along with the spectra of their individual components (*Av* gel, *Av* peel extract and commercial PVP powder), is shown in Figure 10. Detailed characteristic bands of Av were clearly observed in the carbohydrate region (1200–800 cm^−1^), along with bands at 1463 and 1381 cm^−1^ found in both the PVP powder and the electrospun PVP membrane. These bands were also present in the PVP membrane/gel/Av peel extract (8:1.5:1.5). In the spectrum of the gel and lyophilized *Av* peel extract, a band at 1719 cm^−1^ indicated the presence of C-O groups in carbohydrates, suggesting their interaction with PVP in the electrospun membranes and confirming the successful incorporation of *Av* compounds into these membranes.

## 4. Conclusions

In this study, operational conditions were established in the electrospinning process, which led to a decrease in fibers in the electrospun PVP membranes. The processing of the Av plant allowed the bioactive compounds to be obtained and preserved in conjunction with the PVP during the electrospinning of the membranes. The relationship of the PVP polymer and the plant extracts plays an important role because, in our case, it contributed to the considerable decrease in the diameter of the fibers, resulting in the formation of an architecture in the membrane that increases the area of contact between the bioconjugated fibers and the wound, increasing the activity in the compounds that accelerates healing and antibacterial activity due to the incorporation of gel and *Aloe vera* peel extract. Due to the characteristics of our membrane, it would be ideal to evaluate its application in a healing model, as well as its antibacterial activity.

## Figures and Tables

**Table 1:** Electrospinning equipment operating conditions.

Membrane	Applied Voltage (kV)	Feed Rate (mL/h)
A	15	0.2
B	15	0.1

## Data Availability

The original contributions presented in the study are included in the article, further inquiries can be directed to the corresponding author.
